# Intrapartum epidural analgesia and low Apgar score among singleton infants born at term: A propensity score matched study

**DOI:** 10.1111/aogs.13837

**Published:** 2020-03-20

**Authors:** Anita C. J. Ravelli, Martine Eskes, Christianne J. M. de Groot, Ameen Abu‐Hanna, Joris A. M. van der Post

**Affiliations:** ^1^ Department of Medical Informatics Amsterdam University Medical Center Amsterdam the Netherlands; ^2^ Department of Obstetrics and Gynecology Amsterdam University Medical Center Amsterdam the Netherlands

**Keywords:** Apgar score, intrapartum epidural analgesia, national cohort, NICU admission, propensity score matched study, term pregnancy

## Abstract

**Introduction:**

The associations of epidural analgesia and low Apgar score found in the Swedish Registry might be a result of confounding by indication. The objective of this study was to assess the possible effect of intrapartum epidural analgesia on low Apgar score and neonatal intensive care unit (NICU) admission in term born singletons with propensity score matching.

**Material and methods:**

This was a propensity score matched study (n = 257 872) conducted in a national cohort of 715 449 term live born singletons without congenital anomalies in the Netherlands. Mothers with prelabor cesarean section were excluded. Main outcome measures were 5‐minute Apgar score <7, 5‐minute Apgar score <4 and admission to a NICU for at least 24 hours. First, an analysis of the underlying risk factors for low Apgar score <7 was performed. Multivariable analyses were applied to assess the effect of the main risk factor, intrapartum epidural analgesia, on low Apgar score to adjust the results for confounding factors. Second, a propensity score matched analysis on the main risk factors for epidural analgesia was applied. By propensity score matching the (confounding) characteristics of the women who received epidural analgesia with the characteristics of the control women without epidural analgesia, the effect of possible confounding by indication is minimized.

**Results:**

Intrapartum epidural analgesia was performed in 128 936 women (18%). Apgar score <7 was present in 1.0%, Apgar score <4 in .2% and NICU admission in .4% of the deliveries. The strongest risk factor for Apgar score <7 was epidural analgesia (adjusted odds ratio [aOR] 1.9, 95% confidence interval [CI] 1.8‐2.0). The propensity score matched adjusted analysis of women with epidural analgesia showed significant adverse neonatal outcomes: aOR 1.8 (95% CI 1.7‐1.9) for AS <7, aOR 1.6 (95% CI 1.4‐1.9) for AS <4 and aOR 1.7 (95% CI 1.6‐1.9) for NICU admission. The results of epidural analgesia on AS <7 were also significantly increased for spontaneous start of labor (aOR 2.0, 95% CI 1.8‐2.1) and for spontaneous delivery.

**Conclusions:**

Intrapartum epidural analgesia at term is strongly associated with low Apgar score and more NICU admissions, especially in spontaneous deliveries. This association needs further research and awareness.

**Abbreviations**
aOR: adjusted odds ratio
CI: confidence interval
EA: epidural analgesia
LGA: large for gestational age
NICU: neonatal intensive care unit
SGA: small for gestational age

**Key message**

This propensity score matched study showed that epidural analgesia during labor at term is an important risk factor for low Apgar score and for more NICU admissions.

## 1 INTRODUCTION

Most births take place at term and during the last decennia, perinatal mortality at term has become very low in western countries.^1^ In the Netherlands, the risk of birth asphyxia, measured as low Apgar score, among term singletons slightly decreased between 1999 and 2009.^2^ Recent trends are unknown.

A previous study showed that birth asphyxia is the main cause of death in term infants in the Netherlands who were admitted to a Neonatal Intensive Care Unit (NICU).^3^ Therefore, low Apgar score and NICU admission of term born neonates needs more attention for assessment of perinatal health and for research into further optimization of obstetric healthcare. A low Apgar score can result in neonatal mortality and morbidity with long‐term consequences and great social impact for the child and their family.^4^, ^5^, ^6^, ^7^, ^8^, ^9^ In term infants without congenital malformations, a low Apgar score most likely reflects perinatal asphyxia.^6^, ^10^ Underlying risks for asphyxia are hypertensive disorders, diabetes (preexisting and gestational diabetes), non‐cephalic presentation at birth, prolonged second stage of labor and intrapartum epidural analgesia (EA).^6^, ^10^, ^11^, ^12^, ^13^, ^14^ Published data on the impact of EA for pain relief during labor on neonatal outcomes are contradictory. A recent Cochrane review of randomized controlled trials on the effectiveness and safety of EA showed that EA did not appear to have an immediate effect on neonatal status as determined by Apgar scores <7 at 5 minutes or admission to NICU. However, these studies in the review were assessed as low‐quality evidence.^15^ The found associations of EA and low Apgar score in the Swedish Registry might be a result of confounding by indication.^11^ New analysis techniques such as propensity score matching analysis can ameliorate this problem of confounding by indication.^16^ In matching the characteristics of the women who received EA with the characteristics of the control women without EA, the effect of the measured possible confounders is minimized.

In the Netherlands, EA is not generally used (<20%) but it is available on request for pain relief during labor in hospitals. The request for EA has increased over the past years from 15% in 2010 to 19% in 2014 (www.perined.nl).

The aim of this study is to assess the effects of EA on low Apgar score and NICU admission at term among singletons and to analyze underlying risk factors.

## 2 MATERIAL AND METHODS

This study was based on the linked data from the national perinatal registry (PERINED) in the Netherlands.^17^, ^18^ Participation in the registry is obligatory for the practices of midwives, obstetricians and neonatologists, and 98% of all births in the Netherlands are recorded in the registry. Data include information about pregnancy, childbirth and hospital (re)admissions of newborns. External validation of the probabilistic medical record linkage showed that the linkage is of high quality.^17^, ^19^ The data are sent annually to the national registry office, where a number of range and consistency checks are conducted. The data in the perinatal registry are anonymized.

From all singletons born between 1 January 2010 and 31 December 2014 (n = 837 226) we selected all term pregnancies delivered between 37 and 42 weeks (37^+0^ weeks and 42^+6^ weeks) of gestation (n = 786 385). From these 20 801 births (2.6%) were excluded because of congenital anomalies, as were 896 fetal deaths (.1%). Women with prelabor cesarean section were also excluded. The total study population thus consisted of 715 449 pregnancies.

Main outcome measurements are as follows: low Apgar rate, defined as a 5‐minute Apgar score <7 per 1000 liveborn infants, 5‐minute Apgar score <4 and admission to a NICU for at least 24 hours.

### 2.1 Statistical analyses

We investigated risk factors for low Apgar score (<7) and adjusted for general known factors that influence Apgar score. We used maternal age (<25, 25‐34, ≥35 years), parity (nulliparous/multiparous), ethnicity (western/non‐western), socioeconomic status (low, mid, high), gestational age (weeks), presentation at birth (cephalic/non‐cephalic), prolonged second stage of labor (>120 minutes for nulliparous women and >60 minutes for multiparous women, or not prolonged) and small (SGA) or large for gestational age (LGA) (<10th percentile and >95th percentile birthweight related to gender, respectively).^20^ We stratified for mode of delivery and presentation. We tested the possible trend in Apgar <7, Apgar <4, NICU admission and EA with a Cochran‐Armitage Trend test.

We used propensity score matched pair analysis to compare risk groups with and without EA while adjusting for possible indication bias.^16^ This means that for each woman with EA the characteristics of that woman were matched with a comparable control woman without EA. Propensity score matching (in a 1:1 ratio) was utilized to minimize the effects of confounders. The mean propensity scores before and after matching will be compared. Propensity scores of EA were generated with the results of the logistical analysis of the risk factors for EA using the following variables: parity, maternal age, ethnicity, socioeconomic status, hypertension, diabetes (gestational and preexisting), gestational age, SGA, LGA, non‐cephalic birth presentation, start of labor and prolonged secondary stage of labor. The effect of EA on neonatal outcomes after propensity score matching was analyzed both as crude and as adjusted (for factors still significantly different after matching). In addition, a stratified analysis by parity, start of labor and mode of delivery were performed. Statistical significance was set at the .05 level. Data were analyzed using SAS software version 9.4 (SAS Institute Cary, North Carolina, USA) and R statistical software with the Match library for matched pair analysis.

### 2.2 Ethical approval

The committee for research and ethics of Perined approved the study protocol (approval no. 16.13) on 15 April 2016.

## 3 RESULTS

Included were 764 688 singleton liveborn infants at term without congenital malformations. Women with prelabor cesarean section were excluded (49 239), so the study cohort existed of 715 449 women who delivered a term liveborn infant.

Apgar <7 was present in 10.4/1000 liveborn infants, Apgar <4 was present in 1.7/1000 live born infants, NICU admission in 3.7/1000.

The trend in Apgar <7 increased from 9.9/1000 in 2010 to 10.9/1000 in 2014 (*P* = .001); Apgar <4 and NICU admission did not significantly change over the years (*P* = .80).

EA significantly increased with 4% (from 15% in 2010) to 19% in 2014.

In Table 1, risk factors for low Apgar score <7 are described. Multiple regression modeling with Apgar score <7 as the outcome measure showed that EA was the strongest crude and adjusted risk factor for Apgar score <7 (adjusted odds ratio [aOR] 1.91, 95% confidence interval [CI] 1.82‐2.02). In addition, small for gestational age (SGA) <10th percentile (p10), large for gestational age (LGA) ≥95th percentile (p95), nulliparity, non‐cephalic birth presentation, prolonged second stage of labor and delivering at 41 or 42 weeks of pregnancy duration were risk factors for low Apgar score <7. High socioeconomic status was a protective factor for low Apgar score (aOR .93, 95% CI .88‐.98).

**Table 1:** Characteristics for low Apgar score (5‐min <7) in 715 449 term infants

Characteristics	%	Apgar score <7	Apgar score <7			
		Per 1000	Crude odds	95% CI	Adjusted^a^ odds	95% CI
Epidural analgesia	18.0	19.5	2.33	2.22‐2.44	1.91	1.82‐2.02
Non‐cephalic presentation	3.3	24.3	2.48	2.27‐2.70	2.55	2.34‐2.78
Nulliparity	45.8	14.2	1.98	1.89‐2.08	1.72	1.63‐1.81
SGAp10	10.4	16.8	1.75	1.64‐1.86	1.58	1.48‐1.68
Gestational age						
37	7.0	13.5	1.42	1.30‐1.55	1.16	1.06‐1.27
38	14.9	9.7	1.02	0.95‐1.10	0.90	0.83‐0.97
39	25.2	8.4	0.88	0.83‐0.94	0.86	0.81‐0.92
40	31.6	9.5	1.00		1.00	
41	19.6	13.3	1.41	1.32‐1.50	1.28	1.20‐1.36
42	1.8	17.9	1.89	1.65‐2.17	1.44	1.25‐1.66
LGAp95	5.2	13.8	1.35	1.23‐1.48	1.60	1.46‐1.75
Maternal age (y)						
<25	11.7	11.7	1.19	1.11‐1.28	0.95	0.88‐1.01
25‐34	68.5	9.9	1.00		1.00	
>=35	19.8	11.7	1.18	1.12‐1.25	1.32	1.24‐1.39
Non‐western ethnicity	19.0	12.5	1.26	1.19‐1.33	1.22	1.15‐1.29
Hypertension	8.0	15.7	1.59	1.48‐1.70	1.30	1.20‐1.40
Prolonged second stage labor	6.6	12.8	1.25	1.15‐1.36	1.33	1.23‐1.45
Socioeconomic status						
Low	24.6	12.1	1.20	1.14‐1.27	1.14	1.08‐1.20
Mid	50.6	10.1	1.00		1.00	
High	24.8	9.5	0.94	0.89‐0.997	0.93	0.88‐0.98
Diabetes	2.3	15.1	1.47	1.30‐1.67	1.31	1.15‐1.50
Induction of labor	23.0	13.7	1.45	1.38‐1.52	1.10	1.04‐1.17

Abbreviations: LGAp95, large for gestational age ≥95th percentile; SGAp10, small for gestational age <10th percentile.

^a^ Adjusted for all the characteristics in the table.

When we stratified for the intermediate factor mode of delivery, Apgar score <7 showed a higher incidence in instrumental vaginal delivery (25/1000) and secondary cesarean section (32/1000) than in spontaneous delivery (6/1000). However, with EA, the adjusted odds for Apgar score <7 for spontaneous birth (aOR 1.88, 95% CI 1.73‐2.01) was higher than for instrumental vaginal delivery or secondary cesarean section (aOR 1.36, 95% CI 1.24‐1.50 and aOR 1.14, 95% CI 1.04‐1.24, respectively) (Table 2).

**Table 2:** The association of epidural analgesia and Apgar score <7 by mode of delivery

	N	Total No epidural				Epidural				
		Apgar <7		Apgar <7	Apgar <7	Apgar <7	Apgar <7	Apgar <7		
		n	/1000	n/N	/1000	n/N	/1000	Crude odds	Adjusted^a^ odds	
Mode of delivery										
Spontaneous	576 913	3547	6.1	2668	5.4	879	11.1	2.09 (95% CI 1.94‐2.26)	1.88 (95% CI 1.73‐2.03)	
				495 351		78 894				
Instrumental vaginal delivery	73 630	1824	24.8	1065	21.9	759	30.4	1.40 (95% CI 1.27‐1.54)	1.36 (95% CI 1.24‐1.50)	
				48 633		24 997				
Secondary cesarean section	64 906	2100	32.4	1229	30.8	871	34.8	1.13 (95% CI 1.04‐1.24)	1.14 (95% CI 1.04‐1.24)	
				39 861		25 045				
Total	715 449	7471	10.4	4962	8.5	2509	19.5	2.33 (95% CI 2.22‐2.44)	1.91 (95% CI 1.82‐2.02)	
				583 845		128 936				

Abbreviation: CI, confidence interval.

^a^ Adjusted for all the characteristics in Table 1.

In addition, the incidence of Apgar <7 was higher in non‐cephalic presentation. However, with EA the adjusted odds for cephalic presentation with EA was higher (aOR 1.94, 95% CI 1.84‐2.05) than in non‐cephalic presentation (data not shown).

Table 3 shows the effects of EA on low Apgar score <7 before and after propensity score analysis. On the left‐hand columns of the table, the differences in the unmatched study cohort for risk factors for low Apgar score with and without EA are shown. All characteristics of the women without EA differ significantly for women with EA except for prolonged second stage of labor. Women with EA more often tended to be nulliparous, younger, more often have hypertension, were more likely to give birth at 41 and 42 weeks’ gestational age, have less non‐cephalic presentation and were more often induced for labor. Before matching, the mean (SD) propensity score was lower in the non‐epidural group (.17 [.1]) than in the epidural group (.26 [.1]; *P* < .0001).

**Table 3:** Maternal and child characteristics by epidural use for the unmatched and matched cohort

	Before matching			After matching		
	No epidural	Epidural	*P*‐value	No epidural	Epidural	*P* value
	586 513	128 936		128 936	128 936	
Characteristics	%	%		%	%	
Nulliparous	40.3	71.1	<.0001	71.2	71.1	.89
Maternal age (y)						
<25	10.7	16.3	<.0001	16.3	16.3	.77
25‐34	68.9	66.6		66.6	66.6	
>=35	20.4	17.1		17.2	17.1	
Non‐western ethnicity	18.7	20.8	<.0001	20.9	20.8	.52
SES						
Low	24.4	25.5	<.0001	25.7	25.5	.60
Mid	50.7	50.5		50.3	50.5	
High	24.9	24.0		24.0	24.0	
Hypertension	7.0	12.7	<.0001	12.9	12.7	.07
Diabetes	2.1	3.4	<.0001	3.3	3.4	.007
Gestational age (wk)						
37	6.9	7.6	<.0001	7.6	7.6	.93
38	14.8	15.3		15.2	15.3	
39	25.8	22.3		22.1	22.3	
40	32.3	28.4		28.5	28.4	
41	18.7	23.6		23.7	23.6	
42	1.5	2.9		2.9	2.9	
SGA p10	10.1	12.0	<.0001	12.2	12.0	.05
LGA p95	5.3	4.8	<.0001	4.7	4.8	.07
Non‐cephalic presentation	3.6	2.0	<.0001	2.0	2.0	.37
Induction of labor	19.3	39.9	<.0001	39.8	39.9	.85
Prolonged sec. stage labor	6.7	6.6	.22	6.4	6.6	.03

Abbreviations: LGAp95, large for gestational age ≥95th percentile; SGAp10, small for gestational age <10th percentile.

The matched results are shown in the right‐hand columns of Table 2; 128 936 women without EA were selected as control (1:1 ratio) for the 128 936 women with EA.

After matching, the predicted probability of EA in the epidural and non‐EA group was equal (.26 and .26; *P* = .99 after rounding).

In the matched cohort, all risk factors/characteristics of women with EA were comparable to those of the women without EA except for a small difference in women with prolonged second stage of labor, diabetes and SGAp10 (Table 3).

Table 4 showed the main neonatal outcomes after matching. The adjusted odds ratio for epidural on the 5‐minute Apgar score <7 after matching was 1.76 (95% CI 1.65‐1.88). Adjustment for SGAp10, diabetes and prolonged second stage of labor did not change the results. The adjusted odds ratio for EA on low Apgar score <4 was also significantly increased (aOR 1.62, 95% CI 1.37‐1.92) and for EA on NICU admission the aOR was 1.73 (95% CI 1.55‐1.93).

**Table 4:** Epidural analgesia and neonatal outcomes after propensity score matching analysis

	Propensity score‐matched adjusted analysis				*P* value	Epidural analgesia			
	No epidural		Epidural			Odds crude	95% CI	Odds adjusted^a^	95% CI
	128 936	/1000	128 936	/1000					
5 min Apgar <4	220	1.7	356	2.8	<.0001	1.62	1.37‐1.92	1.62	1.37‐1.92
5‐min Apgar <7	1437	11.1	2509	19.5	<.0001	1.76	1.65‐1.88	1.76	1.65‐1.88
NICU admission (>24 h)	497	3.9	855	6.6	<.0001	1.72	1.54‐1.93	1.73	1.55‐1.93

^a^ Adjusted for diabetes, SGAp10 (small for gestational age <10th percentile) and prolonged second stage of labor.

Separate analysis for parity, for start of labor and for mode of delivery showed that for nulliparous women the odds ratio of EA on Apgar score <7 was 1.70 (95% CI 1.58‐1.83) and for multiparous women 2.03 (95% CI 1.75‐2.13). For spontaneous start of labor, the odds ratio of EA on low Apgar score <7 was 1.95 (95% CI 1.79‐2.35) and for induction of labor 1.50 (95% CI 1.38‐1.68) (Table 5).

**Table 5:** Epidural analgesia and low Apgar score (5‐min) <7 by parity, start of labor and by mode of delivery in the propensity score matched cohort

	No epidural		Epidural		Epidural analgesia			
	n/N	/1000	n/N	/1000	Crude odds	95% CI	Adjusted odds^a^	95% CI
Total	1437	11.1	2509	19.5	1.76	1.65‐1.88	1.76	1.65‐1.88
	128 936		128 936					
Parity								
Nulliparous	1160	12.6	1950	21.3	1.70	1.58‐1.83	1.70	1.58‐1.83
	91 760		91 728					
Multiparous	277	7.5	559	15.0	2.03	1.76‐2.35	2.03	1.75‐2.34
	37 176		37 208					
Start of labor								
Spontaneous	788	10.2	1525	19.7	1.96	1.79‐2.13	1.95	1.79‐2.13
	77 548		77 500					
Induction of labor	649	12.6	984	19.1	1.52	1.38‐1.67	1.50	1.38‐1.68
	51 388		51 436					
Mode of delivery								
Spontaneous	633	6.4	879	11.1	1.75	1.58‐1.94	1.74	1.57‐1.93
	98 739		78 894					
Instrumental vaginal delivery	342	20.8	759	30.4	1.47	1.30‐1.68	1.47	1.29‐1.67
	16 469		24 997					
Secondary cesarean section	462	33.7	871	34.8	1.03	.92‐1.16	1.07	.96‐1.20
	13 728		25 045					

Abbreviation: CI, confidence interval.

^a^ Adjusted for diabetes, SGAp10 (small for gestational age <10th percentile) and prolonged second stage of labor.

The risk of EA on AS <7 was higher in spontaneous delivery (aOR 1.74, 95% CI 1.57‐1.93) than in instrumental vaginal delivery (aOR 1.47, 95% CI 1.29‐1.67). Secondary cesarean section carried no additional risk of EA on low AS <7 (Table 5).

## 4 DISCUSSION

The most important risk factor for low Apgar score <7 at term was EA. Other risk factors for low Apgar score <7 were non‐cephalic presentation, SGA < p10, LGA ≥ p95, nulliparity, maternal age ≥35 years and gestational age 41 and 42 weeks.

Propensity score matching for risk factors for EA showed a strong association for EA on low Apgar score <7 and <4 and NICU admissions. The effects of low Apgar score <7 were more pronounced in women with spontaneous start of labor and among spontaneous deliveries.

A strength of our study is that we have used the national perinatal databases cover nearly all births (98%) in the Netherlands.

Another strength is the use of propensity score matching. Women with EA differ in risk factors from women who do not have EA during labor; with propensity score matching, for each woman with EA, a comparable woman without EA is matched, resulting in matched pairs in which the analysis could be performed.

Due to the large study size (715 449) as well as the large propensity score matching population (n = 257 872), the chance of bias is small.

A possible limitation of the study is that we had to base birth asphyxia on the Apgar score <7 because umbilical artery cord pH is not routinely measured in the Netherlands. However, it has been shown that a low Apgar score is a good proxy for birth asphyxia and its relation with neonatal mortality and morbidity has been demonstrated.^5^, ^8^, ^9^, ^10^, ^21^ In our study group of term born singletons without congenital anomalies, low Apgar score is generally caused by intrauterine asphyxia.^10^ Another limitation of this study is the lack of recording of important risk factors for low Apgar score <7 such as cigarette use and body mass index (BMI) in the registry. In addition, intrapartum temperature elevation is not included in Perined, a well‐known complication of EA which is possibly related to adverse outcomes such as birth asphyxia.^22^

Published data on the impact of EA for pain relief during labor on neonatal outcomes are contradictory. A recent Cochrane review of randomized controlled trials on the effectiveness and safety of EA showed that EA did not appear to have an immediate effect on neonatal status as determined by Apgar scores <7 at 5 minutes. However, these studies were assessed as low‐quality evidence.^15^ A population‐based cohort study in Sweden (1988‐1997) among term liveborn infants without congenital anomalies showed a decrease in the rate of low Apgar scores (<7 at 5 minutes) from 7.7/1000 live births in 1988 to 6.3/1000 in 1992 but thereafter an increase to 8.2/10 000 in 1997.^6^ The authors speculated that the increase in multiple births, immigration and EA during 1992‐1997 has contributed to the increase of newborns with 5‐minute Apgar score <7. Another Swedish population‐based study of nulliparous term singletons in 1999‐2008 showed an increased risk of low Apgar score (<7 at 5 minutes) with EA (aOR 1.27, 95% CI 1.16‐1.39). In our study, EA gave a comparable aOR of 1.92 (95% CI 1.82‐2.02) for low 5‐minute Apgar score (<7) and after propensity score matching an aOR of 1.76 (95% CI 1.65‐1.88).

In 2008‐2012, a cohort of all 32 796 nulliparous women in two counties in Sweden who delivered at ≥37 weeks, liveborn singleton infants with cephalic presentation was studied. Of the women, 60% received EA. The total rate of 5‐minute Apgar score <7 was 7/1000; with EA the rate was doubled (8.4/10 000) compared with 4.8/1000 without EA.^12^ In this study, rates of EA increased with longer duration of second stage of labor and this is regarded as a confounder. In our propensity score matched cohort, we found that the significant increased risk for low Apgar score with EA remained comparable to that when adjustment for prolonged second stage of labor was made.

In a case‐control study of late preterm and term infants, Kumar et al found an association between the exposure to EA in labor and the occurrence of respiratory distress.^13^

It is possible that the effect of EA is due to fentanyl affecting infants or the changes in physiology of birth by EA.^23^ It is known from pharmacokinetic studies that fentanyl can diffuse from the epidural space into the maternal blood and across the placenta, and can have an effect on the respiratory center of the neonate.^24^, ^25^

The incidence of EA in the Netherlands is 18%. Our study showed that this increased from 15% to 19% (23%‐31% in nulliparous women). In other countries, a more pronounced increase of EA has occurred over the past 20 years. In the USA, 61% of women who had a singleton birth and vaginal delivery received epidural or spinal anesthesia in 2008 (https://www.cdc.gov/nchs/data/nvsr/nvsr59/nvsr59_05.pdf). In Sweden, 44% of the nulliparous women with singleton pregnancies at term received EA in 1999‐2008.^11^

In the Netherlands, EA is not generally used but it is available on request for pain relief during labor. When low‐risk women are under the care of an independent midwife, transfer to the gynecologist is required when EA is asked for. It is possible that women who choose EA during labor for pain relief differ in their attitude toward medical interventions or have more risk factors for low Apgar score such as nulliparity. Therefore, in our study we performed a propensity score analysis to match risk factors of women who received EA with a control group without EA and, in addition, stratified for parity. The results corroborate the importance of EA on the risk of low Apgar score.

This study showed that EA is the most relevant risk factor for low Apgar score, especially in low risk situations such as spontaneous start of labor, cephalic presentation and spontaneous delivery at term. For daily practice this means when EA is used, more vigilance is needed.

Secondary cesarean section and instrumental vaginal delivery can be indicated when there are signs of (threatening) asphyxia. It is possible that in spontaneous delivery, fetal monitoring too often is lacking, or more expectant management played a role.

Randomized trials are the gold standard to learn more about the pathway of EA in general or the effect of specific drugs on neonatal outcome.^23^ However, when randomization is not possible, a propensity score matched analysis is then an appropriate methodology to apply.

In the Netherlands, where EA during labor is not yet generally applied, there is still an opportunity to perform prospective studies on the effect of EA on the newborn.

## 5 CONCLUSION

EA is an important risk factor for lower Apgar score and NICU admissions at term, especially in women with spontaneous start of labor and spontaneous delivery, and merits further research and awareness.

## CONFLICT OF INTEREST

The authors report no conflict of interest.
